# Realizing Eco-Friendly Water-Resistant Sodium-Alginate-Based Films Blended with a Polyphenolic Aqueous Extract from Grape Pomace Waste for Potential Food Packaging Applications

**DOI:** 10.3390/ijms241411462

**Published:** 2023-07-14

**Authors:** Jennifer Gubitosa, Vito Rizzi, Cosma Marasciulo, Filippo Maggi, Giovanni Caprioli, Ahmed M. Mustafa, Paola Fini, Nicoletta De Vietro, Antonella Maria Aresta, Pinalysa Cosma

**Affiliations:** 1 Dipartimento di Chimica, Università degli Studi “Aldo Moro” di Bari, Via Orabona, 70126 Bari, Italy; vito.rizzi@uniba.it (V.R.); c.marasciulo3@studenti.uniba.it (C.M.); 2 Chemistry Interdisciplinary Project (ChIP) Research Center, School of Pharmacy, University of Camerino, Via Ma-donna delle Carceri 9/B, 62032 Camerino, Italy; filippo.maggi@unicam.it (F.M.); giovanni.caprioli@unicam.it (G.C.); ahmed.mustafa@unicam.it (A.M.M.); 3 Consiglio Nazionale delle Ricerche CNR-IPCF, UOS Bari, Via Orabona, 70126 Bari, Italy; p.fini@ba.ipcf.cnr.it; 4 Dipartimento di Bioscienze, Biotecnologie e Ambiente, Università degli Studi “Aldo Moro” di Bari, Via Orabona, 70126 Bari, Italy; nicoletta.devietro@uniba.it (N.D.V.); antonellamaria.aresta@uniba.it (A.M.A.)

**Keywords:** alginate-based films, grape pomace, polyphenolic extracts, food-packaging, antioxidants, circular economy

## Abstract

Water-resistant and environmentally friendly sodium-alginate-based films have been investigated to develop functional materials to extend the food’s shelf-life. A water-stable alginate-based film was prepared, employing both the internal and external gelation approach in the presence of CaCl_2_. To apply this film to food packaging and thus preserve food quality, the aim of this work is to perform a chemical and physical characterization of the proposed materials, evidencing the main features and stability under different work conditions. Water contact angle measurements showed a value of 65°, suggesting an important reduced hydrophilic character of the obtained alginate films due to the novel CaCl_2_-induced compacted polymer network. The film’s stability was thus checked through swelling measurements in water after varying pH, temperature, and ionic strength. The film was stable at high temperatures and not pH-responsive. Only highly concentrated salt-based solutions negatively affected the proposed packaging, causing a large swelling. Furthermore, a water-based polyphenolic extract from grape (*Vitis vinifera* L.) pomace waste was embedded inside the films in different amounts in order to confer additional properties. The extract’s polyphenolic content (evaluated from HPLC/MS-MS measurements) endowed the films’ UV-light screening and enhanced antioxidant properties. These important findings suggest the additional potential role of these films in protecting food from light deterioration. The stability of these hybrid films was also checked by observation, as the polyphenols’ presence did not largely alter the alginate network that occurred yet was water-resistant under the described work conditions.

## 1. Introduction

Since its discovery, packaging has acquired a key role in the food industry for preserving the quality of many products [1,2]. Polyethylene terephthalate, polyvinyl chloride, polystyrene, polypropylene, and high- and low-density polyethylene are still the main commercially available plastic materials used for packaging foodstuff [2]. Unfortunately, these polymers cannot be considered biodegradable, and they generate a huge amount of waste worldwide each year [3]. One of the consequences is that plastics reach the oceans, negatively impacting aquatic ecosystems. Moreover, the extensive use of plastics favors the depletion of fossil resources [2]. It follows that the use of renewable and biodegradable packaging materials has started to be strongly encouraged, representing the materials of the future in accordance with the European Union Sustainable development strategies [4]. In this context, biopolymers such as chitosan and sodium alginate (SA) are worth mentioning, as they are able to form hydrogels that, if properly dehydrated, induce the formation of free-standing films [3,5,6,7,8,9,10]. Alginate is one of the most used polysaccharides for developing hydrogels because it offers strong gelling and film-forming properties due to the promotion of interchain associations through interactions with external additives (such as polyvalent metal ions). Specifically, SA is an anionic polymer, usually obtained from brown seaweeds [11]. It is composed of linear copolymers with (1→4)-α-l-guluronic acid (G), (1→4)-β-d-mannuronic acid blocks (M), and heteropolymeric sequences of M and G (MG blocks), irregularly arranged according to different proportions of GG, MG, and MM blocks. The ratio between guluronic and mannuronic acid units and block length greatly influences the polymer’s physical and chemical properties [6,12]. Alginate is considered a polyuronide, i.e., a natural ion exchanger, thanks to its affinity for multivalent cations. In particular, alginate shows high selectivity and affinity for divalent cations according to the order Mn^2+^ < Zn^2+^, Ni^2+^, Co^2+^ < Fe^2+^ < Ca^2+^ < Sr^2+^ < Ba^2+^ < Cd^2+^ < Cu^2+^ < Pb^2+^ as a direct function of G unit contents in the polymer [3,6,12].

However, although SA-based materials are largely used in the food industry, they are highly hydrophilic. Indeed, if SA’s films are placed in contact with water, they rapidly swell and dissolve, proving not to be directly applicable as physical barriers for food preservation. For instance, composite and modified materials have been proposed by incorporating nanomaterials, clays, and other additives into SA-based hydrogels. These additives confer the role of smart and active packaging to SA-based materials by boosting their intrinsic properties [11]. Among additives, due to toxicity and other reasons, CaCl_2_ is the most widely used Ca-based salt to prepare the alginate gel. CaCl_2_ can easily promote the simple and fast SA polymer chains’ re-organization into more compacted structures with an extended lifetime in water. Indeed, Ca^2+^ cations, by exchanging sodium ions, can strongly interact with G blocks of the polymer to form bridges between different alginate chains [13,14]. The most popular association is reported to be the “*egg-box model*”, in which the G-blocks form a three-dimensional network, and Ca^2+^ ions are embedded in cavities like eggs in a cardboard egg box. Although a more general model reports that Ca^2+^ ions are localized in different domains, favoring the whole polymer chain’s association [13], the characteristics of Ca^2+^-based alginate gel are linked to the G and M content in the polymer chain. A high G unit content (M/G < 1.0) can form strong, dense, and rigid gels with good thermal stability, while a high M unit content (M/G > 1.0) promotes soft, flexible, and elastic gels with good freeze–thaw properties [12,15]. Interestingly, SA can be crosslinked with Ca^2+^ using several approaches, following two main procedures: the internal and external gelation methods commonly applied to prepare alginate-based beads/pellets [12,16,17,18]. In the case of using external gelation, generally, the alginate solution is dropped into a solution of calcium salt, while in the second method (internal gelation), the SA solution is treated with insoluble calcium salt and then the mix is extruded into a hydrophobic phase. This latter method needs to use an acid solution to induce the release of Ca^2+^ from the insoluble salt and make it available for crosslinking with the alginate [17]. In the case of solid films, the internal gelation approach involves the SA films being prepared by mixing the alginate hydrogel with a CaCl_2_ solution, and the solid-state film formation occurs after the evaporation of water. On the other hand, in the second method, the obtained solid-state SA film is placed in contact with a CaCl_2_ solution after its formation. According to the applications, the two methods would offer SA films made up of polymer chains that are differently packed and water-resistant with respect to the films manufactured without the use of CaCl_2_ [16,17,18].

By starting from these considerations, the aim of this paper is to report a modified protocol for preparing water-resistant SA films by adopting both external and internal gelation methods. Furthermore, to be in line with the recent trends devoted to developing innovative, eco-friendly, and active food packaging [19], a cheap and raw polyphenolic aqueous extract obtained from grape (*Vitis vinifera* L.) pomace waste was embedded into the SA matrix to boost its properties. So, from the perspective of using the proposed films for food packaging applications, for example, in the presence of meat, this work aims to investigate the main physical and chemical features of SA- and SA + GPWW-based films, evidencing their stability under different work conditions.

Moreover, the particularity of the extract used is the simplicity of its preparation, which is directly from wastes, avoiding the use of organic solvent and extraction technologies according to Green Chemistry and sustainability principles [19]. Indeed, the pomace was washed with water to obtain the derived raw Grape Pomace Waste Water (GPWW), offering an option for pomace waste management (the presence of which in the environment could induce harmful effects [20]) and representing another important goal of this work. The proposed alternative reuse would further valorize grape pomace, also considering its high content of polysaccharides, proanthocyanidins, lignin, proteins, and phenols [21]. In this context, the significant presence of the latter bioactive molecules is very interesting due to their many extraordinary associated actions: they are cost-effective, non-toxic, antimicrobial, antioxidant, natural, biocompatible, and biodegradable compounds [22]. These substances are often used in developing active packaging thanks to their efficacy in reducing food spoilage and increasing shelf life [2,23,24,25]. On this ground, this study would additionally investigate the antioxidants and sunscreen properties of the GPWW embedded in alginate-based films for potential food industry applications [26,27,28,29,30,31]. Indeed, a systematic study in the presence of raw GPWW is absent in the literature when referring to SA-based films for food packaging. Only a few examples are reported and devoted to the use of polyphenols derived from plants but not concerning water-resistant SA films [29,32,33,34,35]. Therefore, the characterization and the application of water-resistant SA-based films derived from grape wastes (SA + GPWW) are proposed during this work, demonstrating their stability and properties under different work conditions. Work is in progress in our laboratory to test the proposed films in the presence of beef meat. The obtained results appear very promising, opening a novel horizon in the field of sustainable food packaging.

## 2. Results and Discussion

### 2.1. Physical and Chemical Characterization of SA + GPWW Blended Films

#### 2.1.1. An Overview

**Figure 1:**
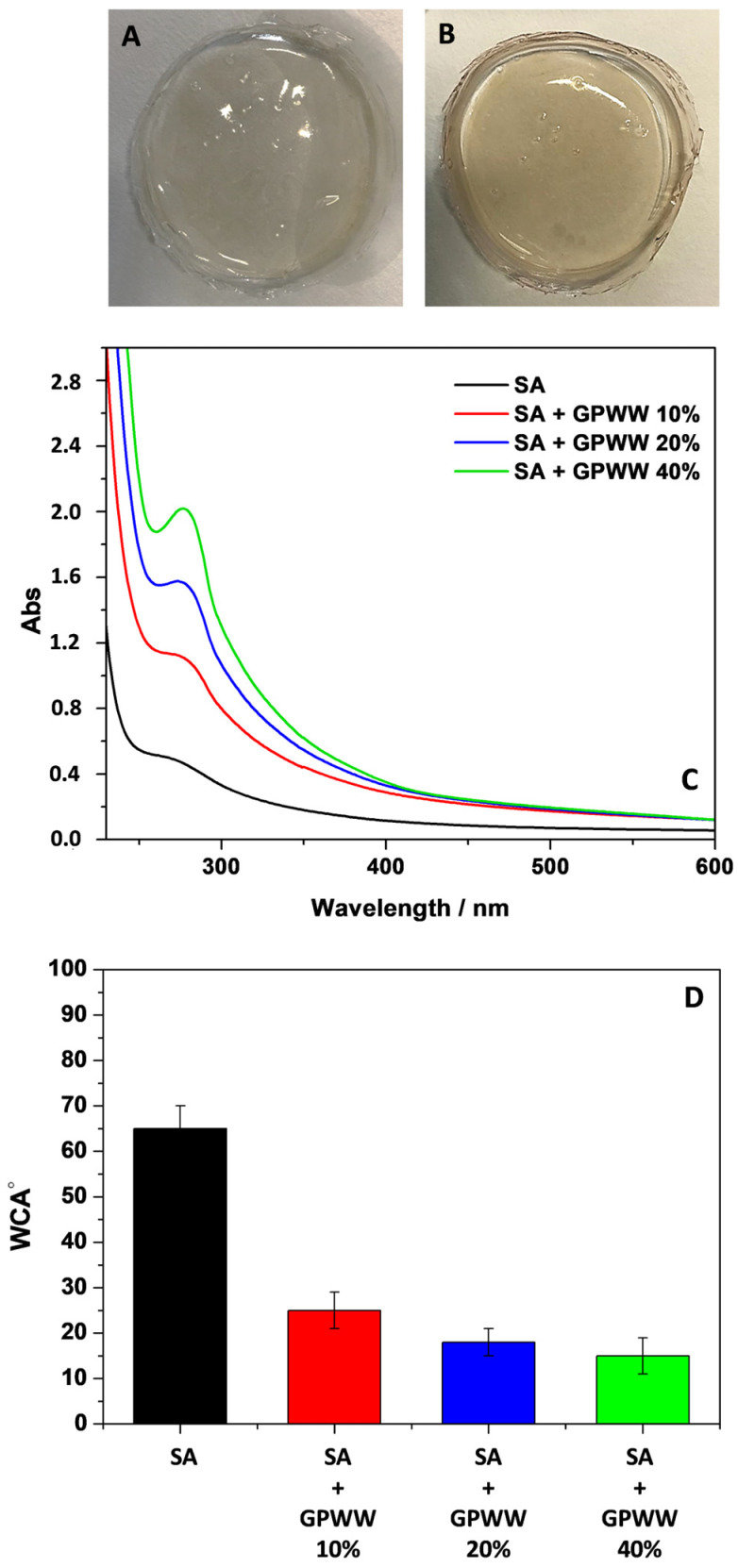
Camera pictures of SA (**A**) and SA + GPWW 40% (**B**) films; UV-Vis spectra (**C**) and WCA measurements (**D**) of SA and SA-based films in the presence of different amounts of GPWW extract: 10, 20, and 40%. The thickness of SA-based films was 1 mm.

As the first step, the SA film obtained by following only the internal gelation method was subjected to investigation to demonstrate the need for both internal and external gelation approaches. Due to its hydrophilic character, this substrate rapidly swelled when placed in water and dissolved in a few minutes. Indeed, when CaCl_2_ is directly mixed with a sodium alginate solution, the reaction is very fast, randomly distributing the crosslinked domains that should be responsible for forming compacted, water-resistant networks [18]. So the obtained solid film should contain high crosslinking density regions dispersed within low crosslinking ones; a film that was not water-resistant was observed. Conversely, if that film, after its formation, was placed in contact with a highly concentrated CaCl_2_ solution (5% *w*/*v*) for a short period (10 min), a water-resistant substrate was obtained and appeared as in Figure 1A.

In this case, two phenomena occurred: (i) hydration and (ii) the further crosslinking reaction with Ca^2+^. Both (i) and (ii) phenomena appeared simultaneously [16]. However, the high CaCl_2_ concentration favored the crosslinking reaction, and the alginate structure collapsed, becoming more compact [16]. The crosslinking domains increased their density, especially at the film surface, which was the first side in contact with the CaCl_2_ solution, packing the polymer chains to form a less permeable surface.

Strong coulombic interactions between Ca^2+^ ions and the alginate carboxylic groups were also expected, compacting the polymer network, and making it less hydrophilic. Overall, the Ca^2+^ cations migrated inside the alginate structure favoring strong inter- and intra-molecular bonds, reducing water channels; consequently, the SA films appeared less permeable to water.

Accordingly, the obtained SA film changed its color from transparent to whitish, due to the novel Ca^2+^-mediated entanglement of alginate chains [16]. Wettability and WCA measurements were thus performed (Figure 1D), confirming this finding. This kind of SA film showed a WCA value of about (65 ± 5)°, which indicated the novel arrangement of the polymer film surface with a lower hydrophilic character with respect to SA film without further modification [36] that, as earlier discussed, dissolved completely in water.

To confer to SA additional properties, i.e., antioxidants and UV-light screening behavior to protect food, GPWW was embedded inside the SA network in different aliquots, 10, 20, and 40% (*v*/*v*). This implies films with increasing amounts of polyphenols derived from GPWW, which showed a total phenolic content of 149.73 mg/L. Specifically, the extract analytical composition was obtained using HPLC/MS-MS measurements, and Table 1 reports the results. Polyphenols with a different chemical nature were detected, and their quantities are expressed in mg/L.

Interestingly, after GPWW and SA hydrogel were mixed, a homogeneous pinkish blend was observed due to the GPWW’s presence. After the drying process and the contact with the CaCl_2_ solution, the color was retained, and the films appeared homogeneous (Figure 1B). This last aspect was important to highlight that the hybrid substrates did not show the macroscopic segregation of SA and GPWW, suggesting the compatibility of the two components. Indeed, the films appeared smooth and flat without cracks and irregular domains. Furthermore, no difference in transparency could be visually observed between pure alginate films and those containing GPWW.

However, WCA measurements (Figure 1D) suggests a novel surface arrangement of the alginate network showing a more hydrophilic surface due to the introduction of GPWW [36]. The WCA passed from 70 °C to 25 °C, 20 °C, and 18 °C according to the increased amount of GPWW in SA’s films. The addition of GPWW increased the wettability, probably due to the contribution of the introduced hydrophilic moieties. Specifically, GPWW contained water-soluble polyphenols, exhibiting hydroxyl groups that should favor the superficial interaction of the composite films with water. Moreover, GPWW was an aqueous extract, and obviously, its addition to the alginate hydrogel decreased the overall solid content of the film in favor of more hydrophilic structures. Indeed, the main components of GPWW should form novel interactions with alginate by weakening the structure [37].

However, as discussed in the following sections, the hybrid films appeared stable in water for a long time, like the SA films, enabling their use in potential food packaging applications.

#### 2.1.2. UV-Vis and ATR-FTIR Measurements

**Figure 2:**
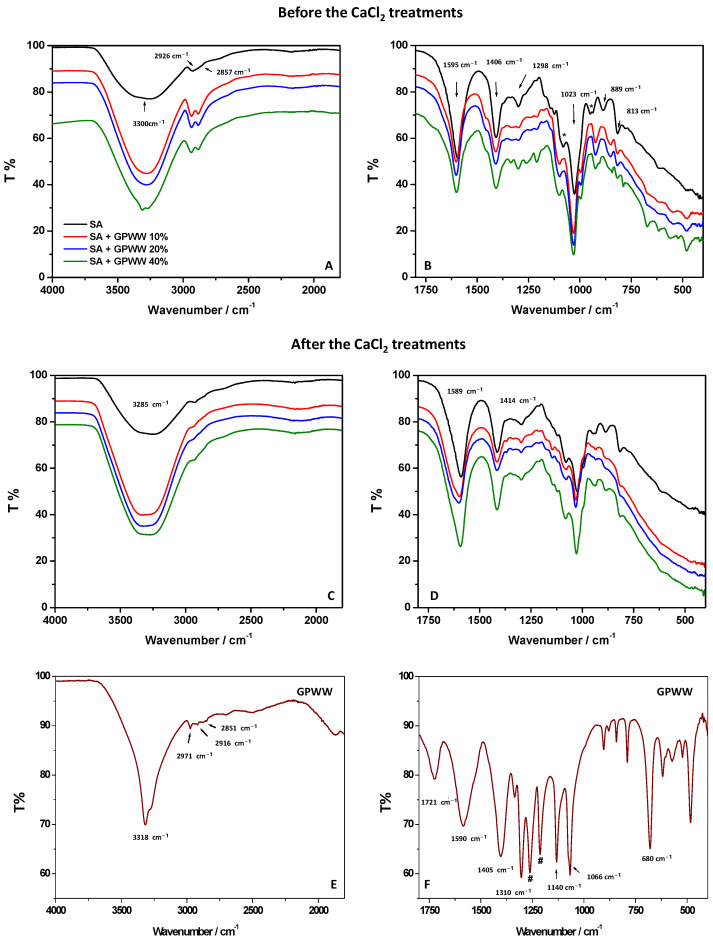
Different wavenumber regions (4000–1800 cm^−1^ and 1800–400 cm^−1^) in the ATR-FTIR spectra and UV-Vis spectra of SA, GPWW, and SA + GPWW-based films in the presence of different amounts of extract: 10, 20, and 40%.

Besides the change in color and WCA passing from SA to SA + GPWW films, interesting information was observed during the spectroscopic investigation performed to assess the role and effect of GPWW. In particular, the contribution of GPWW, according to its amount, was progressively appreciated during the UV-Vis analysis (Figure 1C). If the SA film did not show a significant absorption in the UV-Vis range, the SA + GPWW composite films exhibited an increased absorbance intensity (λ < 350 nm), directly correlated with the % GPWW [20]. The signal intensity increased when passing from SA + GPWW 10 to 40%. No important differences were detected in the positions and shape of bands when comparing samples obtained before and after the contact with the CaCl_2_ solution. On the other hand, differences were observed during the ATR-FTIR analysis (Figure 2). Two wavenumber regions (4000–1800 and 1800–500 cm^−1^), reported in Figure 2, were separately investigated to better appreciate the signals.

Starting from the SA film obtained with the internal gelation approach (Figure 2A,B), the FTIR spectrum showed characteristic and typical signals of alginate: a broad band at 3300 cm^−1^ ascribed to the stretching of hydrogen-bonded –OH groups; a band at 2926 cm^−1^, attributed to -CH_2_ symmetric and asymmetric vibration; and the alkyl groups at 2857 cm^−1^ [38,39,40,41]. The bands at 1595 and 1406 cm^−1^ can be attributed to the symmetric and asymmetric vibrations of the carboxylate moieties (COO^−^). At 1023 cm^−1^, C–O–C stretching was clearly observed due to the alginate saccharide structure. Small peaks were detected at 1088 and 947 cm^−1^ (indicated with asterisks, *) and derived from CO groups coupled with the stretching vibrations of the pyranoside rings (deformation of C–C–H and C–O–H). The signals retrieved in the 900–620 cm^−1^ range (at 889 cm^−1^ and 813 cm^−1^) are characteristic vibrations of mannuronic acid residues.

After the treatment with CaCl_2_ (external gelation approach), if the whole FTIR profile of SA film was retained, some important changes were observed (Figure 2C,D). The OH stretching moved from 3300 to 3285 cm^−1^. The bands at 1595 and 1406 cm^−1^, ascribed to the symmetric and asymmetric vibrations of the carboxylate moieties (COO^−^), shifted to 1589 and 1414 cm^−1^, respectively. The other signals below 1088 cm^−1^ retained their shape and wavenumber position. These findings can be attributed to the Ca^2+^-induced effect during the external gelation [42]. Not surprisingly, the observed FTIR changes, mainly attributed to the two peaks at 1595 and 1406 cm^−1^, are reported to be diagnostic in the literature to investigate the Ca^2+^ crosslinking process. It is well known that when the SA film is crosslinked with Ca^2+^, the asymmetric COO^−^ vibrational peak shifts at lower wavenumbers, and the symmetric one shifts towards higher wavenumbers. This behavior was clearly observed in this work, evidencing the presence of strong electrostatic interaction between Ca^2+^ and carboxylic groups of the polymer that strongly packed its structure. Ca^2+^ rendered the film less hydrophilic with the consequence of significantly affecting its surface wettability in accordance with WCA measurements [18,42]. To better understand the effect of GPWW on the SA network, SA + GPWW composite substrates were also analyzed (Figure 2A–D) with ATR-FTIR before and after contact with CaCl_2_. Moreover, to understand the contribution of GPWW when mixed with SA, the FTIR spectrum of GPWW was also acquired (Figure 2E,F). In agreement with HPLC-MS/MS analysis (see Table 1), the FTIR analysis of GPWW confirmed the prominent presence of polyphenols in the extract [19]. Indeed, a sharp band centered at 3318 cm^−1^ and attributed to OH vibration was observed, together with a signal at about 2900 cm^−1^ ascribed to C-H groups. The CH_3_ out-of-plane bending and scissoring were also observed at 1370 and 1310 cm^−1^ (indicated with **#**), respectively. Signals at 1143 and 795 cm^−1^ corresponding to aromatic C-H stretching and rocking of CH_2_ of phenolic compounds were also detected. If the band at 1721 cm^−1^ was attributed to C=O groups, on the other hand, the signal around 1590 cm^−1^ was associated with the aromatic C-C stretching of polyphenols [19]. Accordingly, at around 1600 cm^−1^, a vibration, usually attributed to the C=C–O deformation of the heterocyclic C-rings, was revealed. The bands between 1405 and 1066 cm^−1^ can be associated with C-H scissoring and bending of the alkane group, and C-O stretching of alcohols, ethers, carboxylic acid, and ester groups are in the 1150–1050 cm^−1^ range [18].

When GPWW was blended with SA, some changes were observed (Figure 2A,B). The contribution of GPWW appeared in the whole spectrum and was more evident, especially when the highest amount of GPWW (40%) was used. In particular, the band at 3300 cm^−1^ was sharper compared with the one from SA, and at the same time, the contribution at 2900 cm^−1^ was more defined. Signals due to the superposition of GPWW bands were also evident in the 1500–900 cm^−1^ region. Interestingly, the band of SA at 1595 cm^−1^ shifted toward higher wavenumbers in SA + GPWW composites. This result does not depend on the overlap of the GPWW signals at the same position (1590 cm^−1^). Indeed, in this latter case, the signals should move toward lower wavenumber values. Conversely, the finding can be attributed to the presence of electrostatic interactions and H-bonding between GPWW and COO^−^ moieties of SA [18]. Additionally, the signal at 1023 cm^−1^ shifted at higher wavenumber values, and this evidence can be associated with the partial overlap of the GPWW signal at 1066 cm^−1^ and the interaction of GPWW with the alginate saccharide structure.

The shift toward lower wavenumbers of bands below 1000 cm^−1^ (attributed to mannuronic acid residues) confirms the previous observation [18]. So GPWW, when mixed with SA, affected the polymer network, creating new interactions and disturbing the previous. The presence of OH moieties from polyphenols should re-arrange the system, weakening the alginate network. Indeed, this effect was more evident when ATR-FTIR measurements were focused on films obtained with external gelation (Figure 2C,D). The presence of GPWW altered, to the same extent, the SA network, as previously described for the SA + GPWW films obtained with the internal gelation method [43]. However, in this case, the shift of the carboxylic bands after the addition of GPWW was more evident due to the effect of GPWW on more strongly packed structures. Nevertheless, as previously said, the composites were still stable in water, meaning that the changes attributed to the GPWW additions did not significantly alter the bulky features of the alginate films. Thus, in order to confirm this assessment, the % of swelling in water, as a massive event involving the whole film and not only the surface wettability, was deeply investigated.

### 2.2. Swelling Measurements

**Figure 3:**
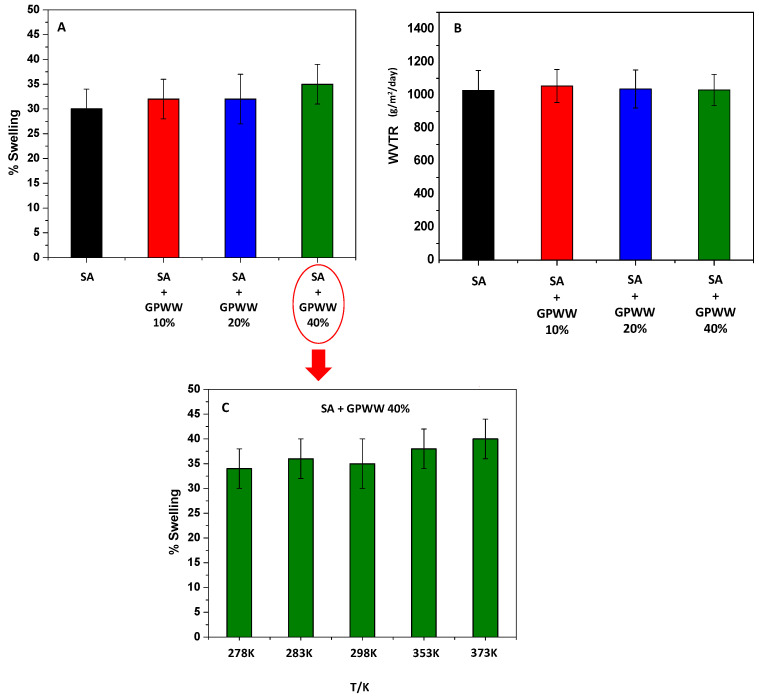
% of swelling (**A**) and WVTR analysis (**B**) related to SA- and SA + GPWW-based films at 10, 20, and 40% in water. Effect of temperature on the swelling of SA + GPWW 40% (**C**).

The water resistance of SA-based films was tested by measuring their stability under extreme working conditions after immersing them in water; the swelling behavior and the effects of several parameters were thus evaluated. Specifically, dried films were placed in distilled water until their weight remained constant, and Equation (1) was applied. So, by fixing the pH at 6 and the temperature at 298 K, SA, and SA + GPWW films were compared under this condition. As previously mentioned, if SA without the external gelation treatment swelled rapidly and in 5 min dissolved in water, on the other hand, SA immersed in CaCl_2_ showed a very low % of swelling (Figure 3A), which is an important feature for the proposed application. Interestingly, although the WCA decreased, meaning an increased hydrophilic surface of SA-GPWW films, the swelling value of ~30% remained almost constant even after GPWW was added. If a trend in the measurements from SA− to SA + GPWW-blended films could be observed, the differences cannot be considered significant, and only in the case of SA + GPWW, in which the GPWW extract was added at 40%, the % of swelling appeared slightly increased. Overall, this low % of swelling could be mainly attributed, as previously explained, to anionic carboxylic moieties in SA, which, after the treatment with CaCl_2_, collapsed due to strong inter- and intra-molecular bonds within the alginate matrix, rendering the SA films less permeable to water [44].

Conversely, the addition of GPWW, due to the large presence of OH moieties, altered the alginate network and favored the interaction with water, slightly increasing the % of swelling [6,45].

However, the swelling of the SA + GPWW films can still be considered low, confirming that the bulk properties were retained.

WVTR analyses were also performed to confirm this finding and obtain more information. Indeed, WVTR evaluates the moisture barrier ability of the materials. According to Figure 3B, the SA and SA + GPWW composite films exhibited the same moisture transmittance performance, so the compacted internal alginate network induced by CaCl_2_ was so strong that it was also retained after the addition of GPWW.

After this assessment, the film was stressed and placed in water at different temperature values ranging from 278 K to 373 K to demonstrate its high stability. The results reported in Figure 3C show that the temperature changes did not significantly affect the swelling degree. The swelling of the SA + GPWW substrate was observed only at the highest tested temperature, probably due to its lower WCA, which enhances bulk hydration under this condition.

For comparison, SA and SA + GPWW 40% were subjected to additional investigation to further study the hybrid films’ features. The swelling measurements were subsequently performed under different conditions to evaluate the role of pH and ionic strength.

#### Effect of pH and Ionic Strength on the Degree of Swelling

Although the swelling of alginate, due to its chemical features, is expected to be pH-dependent [46], the proposed packaging material appeared not to be responsive to the change in pH values (Figure S1A,B). This is an important feature in the field of the proposed application. Indeed, during food deterioration, the growth of microorganisms and biogenic amines is favored, and the pH of the food could be altered [47]. So a material that is stable in a large pH range should be preferable to extend the food’s shelf life. Usually, it is reported that the pK_a_ values of mannuronic and guluronic acids of alginate are 3.38 and 3.65, respectively. At pH < pK_a_, the carboxylic moieties are protonated, and the alginate structures should be in a collapsed state. On the other hand, at pH > pK_a_, the deprotonation of COOH starts, and the repulsion between COO^−^ groups occurs, favoring the swelling [46].

**Figure 4:**
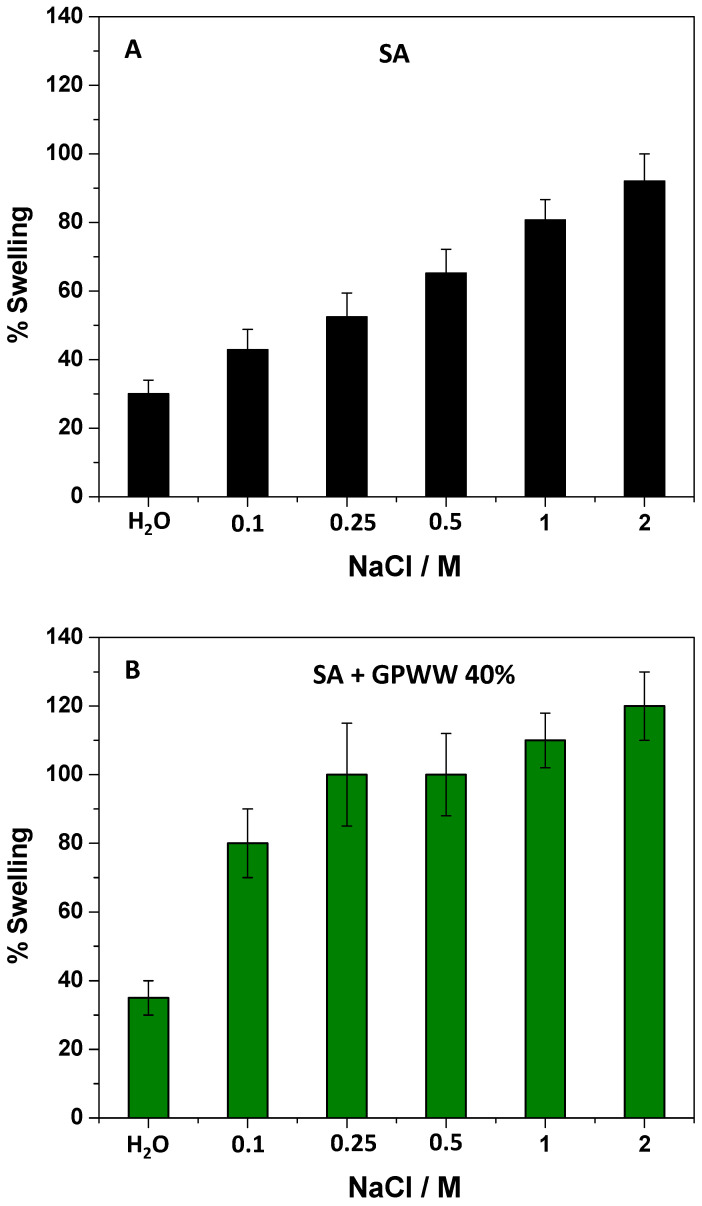
% of swelling related to SA (**A**) and SA + GPWW 40% (**B**) in water at different NaCl concentrations.

During this work, the further external gelation of SA films strongly crosslinked the alginate network, so its hydration occurred to a minor extent. The result was that the presence of H^+^ or OH^−^ in water did not perturb the alginate skeleton. More importantly, although the presence of GPWW extract perturbed the SA network, it did not affect this behavior (Figure S1B), and SA + GPWW films were still not pH-responsive. Instead, with respect to these results, the presence of electrolytes greatly affected the swelling of the proposed films (Figure 4 and Figure S2). To demonstrate this assessment, NaCl was adopted as the model salt, and the films were placed in contact with water solutions with different salt concentrations ranging from 0 to 2 M. The effect was at first evaluated on SA films (Figure 4A). As expected, the presence of salt favored the ion exchange between the Na^+^ present in water and the Ca^2+^ embedded in the alginate network, weakening the assembly [48]. Zhang et al. [49] reported that Ca^2+^ ions linked to COO^−^ units and, under this condition, started to absorb more water and Na^+^ ions, which replaced Ca^2+^. The result was that Ca^2+^ ions appeared in a highly hydrated egg-box structure and diffused from the film into the solution. Then, the egg-box structures were loosened, favoring the water adsorption into macromolecular chains, and finally, the film appeared disassembled [49]. This clearly depended on the higher solubility of the sodium alginate in aqueous media than calcium-alginate. Indeed, swelling and solubilization were more evident when the amount of NaCl was high and the % of swelling reached 100%. Macroscopically, the film tended to assume a hydrogel consistency. This effect appeared more pronounced when SA + GPWW films were considered (Figure 4B), confirming the weakness of SA + GPWW structures with respect to SA when saline solutions were used. In particular, the SA + GPWW 40% behavior is reported in Figure 4B as a model, but the other SA + GPWW films behaved similarly.

Due to the presence of GPWW, the % of swelling started to increase already at lower NaCl concentrations: the more hydrophilic surface enhanced the hydration by favoring the swelling. Moreover, by further increasing the amount of NaCl from 0.25 to 1 M, the % of swelling was levelled off at around 100%, and then it increased again until about 120% at NaCl 2 M.

Afterward, the swelling % was calculated in the presence of different electrolytes to study the effect of salt and thus evaluate the application field. More specifically, the nature of cations and anions was changed, fixing the concentration of electrolytes at 0.5 M (Figure S2).

Starting with SA films, the obtained results are reported in Figure S2A. In particular, LiCl, NaCl, KCl, MgCl_2_, and CaCl_2_ were employed to investigate cations’ role by fixing the nature of the anion (Cl^−^). By changing the cation size from Li^+^ to K^+^, the % of swelling increased from 40 to 100%. The exchange between K^+^ and Ca^2+^ was probably favored, observing the rapid destruction of the Ca^2+^-based alginate network. This behavior could be justified by considering the size of cations, the related hydration degree, and their polarizability. For example, the following hydration radii are reported: K^+^ = 2.32, Na^+^ = 2.76, and Li^+^ = 3.4 Å [50]. So the K^+^ exchange with Ca^2+^ was probably favored with respect to the others due to its smaller size. When bivalent cations were studied, the effect was more pronounced. Indeed, the use of MgCl_2_ rapidly broke the SA structure, favoring its quite complete swelling. Due to its nature, Mg^2+^ efficiently competes with Ca^2+^ by replacing it in the SA network and enhancing the disruption. Indeed, it has been reported that Mg^2+^ is considered a non-gelling ion or an ion with a very slow kinetic in the formation of the alginate hydrogel that, in any case, is considered not stable in a water medium [51]. On the other hand, when Ca^2+^ was adopted, as expected, the SA structure was not disturbed, swelling similarly to what was observed in water.

Regarding the composite films, a similar behavior was observed (Figure S2B). In this case, the interrupted crosslinked structure was associated with the presence of GPWW; since it increases the films’ wettability, it renders the effect of cations Li^+^, Na^+^, and K^+^ more evident, even if the cations appear to level off the swelling percentages without significant differences. This behavior depended on a process starting at the surface of a more hydrophilic film, weakening the structure and favoring the increment of swelling. For example, for the Li^+^ case, the % of swelling was shown to be almost double that observed for SA. Although the change in monovalent cations did not significantly affect the swelling %, passing to divalent ions, the film’s swelling property again changed greatly. This was particularly true when Mg^2+^ was used, with a swelling percentage 6-fold times greater than in water, while the presence of Ca^2+^, as previously stated, did not affect the SA + GPWW films swelling in water.

Therefore, overall, the use of the proposed packaging should be avoided in the case of foods that are particularly rich in salts, except for those containing Ca^2+^, which should further stabilize the SA-based material. However, it is worth mentioning that the experiments simulated very hard work conditions with materials immersed in a water solution.

### 2.3. Antioxidant Features and the Photostability of SA and SA + GPWW Films

#### 2.3.1. Antioxidant Activity

**Figure 5:**
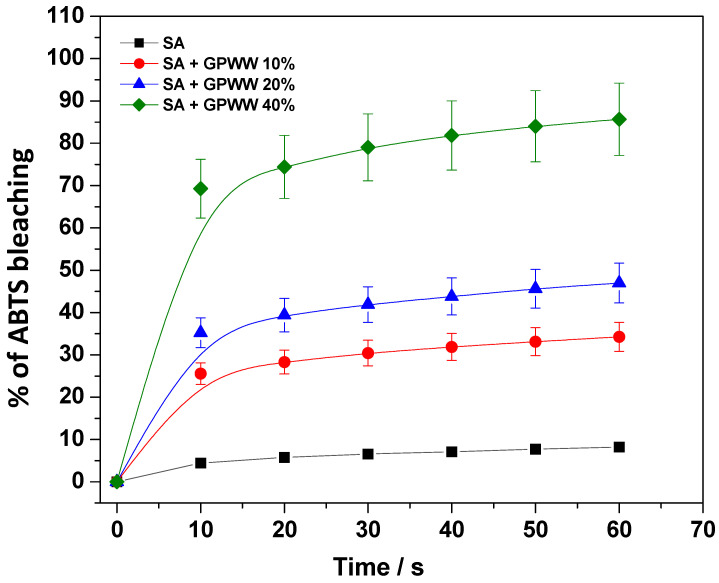
% of ABTS bleaching when in the presence of SA and SA + GPWW 10, 20, and 40%.

Due to the antioxidant properties of the GPWW extract, the antioxidant ability of both SA and SA + GPWW films was evaluated by employing the ABTS assay. More specifically, the experiments were carried out to demonstrate that GPWW also retained its antioxidant activity after the blend with SA film, boosting the action of SA. Specifically, to simulate a situation during which the film could exert its maximum activity, the external treatment with the CaCl_2_ solution was avoided. Thus, its dissolution in water was obtained. To compare the activity of each type of studied film (SA, SA + GPWW 10%, 20%, and 40%), a suitable amount of these aqueous solutions was diluted in PBS, 0.1 M, pH 7.5, in the presence of ABTS, and spectrophotometrically monitored. Equation (2) was thus used to infer the % of ABTS bleaching. The results are reported in Figure 5, where the clear dose-dependent effect of GPWW can be observed. If the SA films, under the conditions explored in work, showed very low antioxidant activity, on the other hand, the SA + GPWW blended films induced an important bleaching of ABTS, indicating a strong antioxidant action: the ABTS was bleached in a few minutes. In particular, in the case of SA + GPWW 40%, 70% of the bleaching of ABTS was reached in 10 min, and then the percentage leveled off at around 90%. So the proposed materials have a great potential to fight oxidative stress and preserve food quality for an extended time.

#### 2.3.2. UV-Light Protective Role of GPWW in SA + GPWW Composite Films

The presence of polyphenols and the associated antioxidant activity suggest another characteristic of these films: their potential feature of screening light irradiation, particularly in the UV range, which is harmful to foods. As reported in Figure 1C, the SA + GPWW films show an intense absorption in the UV region of the electromagnetic spectrum due to the presence of polyphenols. In fact, their property of shielding the irradiation of light has been well-known for many years [52]. Therefore, the presence of GPWW in SA could have the potential to protect food from UV light by absorbing it. Indeed, in many supermarkets, the food is packed and placed in the fridge, being continuously exposed to light sources. In particular, light energy in the ultraviolet and visible light regions has an important role in overall food quality, leading to various degradation and oxidation reactions. The destruction of nutrients and bioactive compounds, the formation of odors and flavors, the loss of food color, and the formation of toxic substances could occur, reducing the shelf-life and nutrition quality [53,54]. Moreover, it has been reported that alginate is destroyed by UV light, and the presence of GPWW could potentially extend its stability by absorbing the irradiation. As a result, the use of packaging materials that are stable under any type of light irradiation conditions and, at the same time, able to fight oxidative reactions should be advisable to preserve food quality [55]. The proposed innovative composite packaging material meets these requirements. It not only exhibits antioxidant activity but, as reported in Figure S3, could be considered photostable under irradiation with light with different energy. For this purpose, the films were exposed to light irradiation deriving from different sources, such as a solar simulator lamp, a neon lamp, and a UV lamp, assessing their stability spectrophotometrically after several irradiation times. In particular, the absorbance intensity (A) at 280 nm was diagnostic to follow the possible bleaching of SA and polyphenols in GPWW. Normalized results with respect to the absorbance intensity read at time zero (A_0_) are reported in Figure S3. By starting from visible light irradiation obtained using a neon lamp, both SA and SA + GPWW composite films were shown to be stable without observing any spectroscopic changes in the bands’ position and intensity. On the other hand, the use of a solar simulator lamp slightly affected the stability of SA due to the UV light component in the solar spectrum. Indeed, SA is susceptible to UV light because it absorbs energy from UV wavelengths, favoring the formation of free radicals, which subsequently cleave the glycosidic bonds, destroying the SA network [56,57]. On the other hand, when the SA + GPWW composite film was studied, a certain photostability was observed. The results can be rationalized by considering that polyphenols can act (i) as a sunscreen material, absorbing the UV radiation, and (ii) as antioxidants, scavenging the UV-induced radical formation, thus preventing the whole SA degradation. These effects were more pronounced when the films were irradiated with UV light. Indeed, while the SA film is clearly affected by this irradiation, the presence of GPWW appears to protect the SA from direct UV-light damage, demonstrating its additional and potential role in preserving the packaging material and the food.

## 3. Materials and Methods

### 3.1. Chemicals

#### 3.1.1. Reagents for the Preparation of SA and SA + GPWW Films

Alginic acid sodium salt from brown algae (medium viscosity); CaCl_2_ anhydrous, granular, ≤7.0 mm, ≥93.0%; glycerol (anhydrous, reagent grade, having ≥99.5% purity); ABTS (2,2-Azino-bis (3-ethylbenzothiazoline-6-sulfonic acid) diammonium salt); (NH_4_)_2_S_2_O_8_, reagent grade, 98%; NaCl anhydrous, Redi-DriTM, ACS Reagent, ≥ 99%; NaOH (Sodium hydroxide), reagent grade, ≥98%, pellets (anhydrous); NaBr, BioXtra, ≥99%; KCl, BioReagent, suitable for cell culture, suitable for insect cell culture, ≥99%; LiCl, Pure Reagent, RPE-ACS; NaI, ACS Reagent, ≥99.5%; NaClO_4_, ACS Reagent, ≥98.0%; MgCl_2_, BioXtra, ≥99%; and HCl, ACS reagent, 37%, were purchased from Sigma-Aldrich (Milan, Italy).

#### 3.1.2. Reagents and Standards for HPLC Analysis

PhytoLab provided analytical standards for kaempferol-3-glucoside and quercetin-3-glucoside (Vestenbergsgreuth, Germany). Sigma-Aldrich (Milan, Italy) provided the reference materials for the remaining 36 of the 38 analytes investigated. Stock solutions of each analyte (1000 mg L^−1^) were made by dissolving pure reference materials in methanol (HPLC-grade) and then keeping them at 5 °C in glass stoppered bottles until analysis. Solutions of working standards at different concentrations were made fresh by diluting stock solutions with methanol (HPLC-grade). Merck (Darmstadt, Germany) provided formic acid at a concentration of 99%. Methanol of HPLC quality was acquired from Sigma-Aldrich. A Milli-Q SP Reagent Water System (Millipore, Bedford, MA, USA) was used to filter deionized water to give ultrapure water with a resistivity of >18 M cm. Sartorius Stedim provided 0.2 μm polyamide filters which were used to filter all liquids (Goettingen, Germany). Phenex™ RC 4 mm 0.2 μm syringeless filters purchased from Phenomenex (Castel Maggiore, BO, Italy) were used to filter all samples before injection into the HPLC instrument.

### 3.2. Grape Pomace Wastes

Grape pomace wastes were received from a local supplier, L’Archetipo (Contrada Tafuri sp 21, km 7, Castellaneta, Taranto/Puglia 74,011 (Italy)), and stored at −19 °C before use. Distilled water was used to prepare aqueous solutions and obtain the polyphenolic extract.

#### GPWW Obtained from Grape Pomace Waste

GPWW was prepared by adding 50 g of as-received mixed grape waste (seeds, skin, and stems) into 1500 mL of distilled water and boiling it for 30 min. Subsequently, to remove the coarse solid residual, vacuum filtration was accomplished. Then, the derived aqueous extract was centrifuged with a Thermo Scientific Heraeus Multifuge X3R Centrifuge and stored at −19 °C before its use. The analytical characterization of the obtained extract was performed using HPLC-MS/MS analysis. The quantification of 38 bioactive analytes belonging to different classes of polyphenols was carried out using a modified version of the already-known method presented by Mustafa et al. [36], as detailed below.

### 3.3. HPLC-MS/MS Analysis

The HPLC-MS/MS investigations were carried out with an Agilent 1290 Infinity series and a Triple Quadrupole 6420 from Agilent Technology (Santa Clara, CA, USA) linked to an electrospray ionization (ESI) source that operated in negative and positive ionization modes. Using Optimizer Software, each standard’s MS/MS parameters were optimized using flow-injection analysis (FIA). The separation of phenolic compounds was obtained through direct injection of diluted GPWW (1:5), using gradient elution mode on a Phenomenex Synergi Polar–RP C18 column (250 mm × 4.6 mm, 4 µm), employing a mixture of water and methanol as solvents A and B, respectively, both with 0.1% formic acid. A Polar RP security guard cartridge preceded the column (4 mm × 3 mm ID) for column protection. The mobile phase composition was made up of the following components: 0–1 min, isocratic condition, 20% B; 1–25 min, 20–85% B; 25–26 min, isocratic condition, 85% B; 26–32 min, 85–20% B. A 0.2 μm polyamide filter was used to filter all solutions and solvents. The injection volume was 2 μL, and the flow rate was kept at 0.8 mL/min. The column temperature was set to 30 °C, and the drying gas temperature in the ionization source was set to 350 °C. The flow rate of the gas was set to 12 L/min, the capillary voltage was 4000 V, and the nebulizer pressure was 55 psi. The peak areas were integrated for quantitation after detection in the dynamic–multiple-reaction-monitoring (dynamic-MRM) mode. Each analyte’s most abundant product ion was employed for quantification, while the other ions were used for qualitative analysis. Each compound’s unique time window (Δ retention time) was set at 2 min.

### 3.4. Design and Preparation of SA and SA + GPWW Films

The SA hydrogels (1% *w*/*v*) were prepared by solubilizing the alginic acid sodium salt powder in distilled water. Then, glycerol (1 mL/100 mL), as a plasticizer, and CaCl_2_ (2.5% *w*/*v*) were added. The derived mixture was stirred for 24 h at room temperature to ensure the complete dissolution of alginate. The investigated films were prepared to start from this system by evaporative drying, pouring the obtained hydrogel into a plastic Petri dish (while avoiding bubble formation). As the next step, the films were dried in an oven at 60 °C for 24 h. By following this approach, free-standing, water-soluble solid films were obtained. So the procedure was slightly modified to obtain films that were more resistant to water. In detail, after their formation, the free-standing solid films were soaked with a CaCl_2_ 5% (*w*/*v*) solution for 10 min. In this way, SA solid films that were not water-soluble and that had a thickness of 1 mm, measured with a caliper micrometer, were obtained.

The same procedure was applied to prepare composite films containing the GPWW. The only difference is the addition of different amounts of GPWW into SA hydrogel. Three different GPWW concentrations (10, 20, and 40% (*v*/*v*)), corresponding to SA + GPWW 10%, SA + GPWW 20%, and SA + GPWW 40%, respectively, were used.

### 3.5. UV-Visible Measurements

The UV-Visible absorption spectra were collected in the 200–800 nm range, at a 1 nm/s scan rate, using a Varian CARY 5 UV-Vis-NIR spectrophotometer (Varian Inc., now Agilent Technologies Inc., Santa Clara, CA, USA). Measurements on solid samples were performed by using an appropriate cell holder.

### 3.6. ATR-FTIR Spectroscopic Measurements

ATR-FTIR spectra were recorded within the 400–4000 cm^−1^ range using a Fourier Transform Infrared spectrometer (FTIR Spectrum Two from Perkin Elmer, Waltham, MA, USA), whose resolution was set to 4 cm^−1^. Sixteen scans were summed for each acquisition.

### 3.7. Swelling Measurements

Square-shaped (1 cm × 1 cm) SA and SA + GPWW films were accurately weighted (W_0_) and placed in a water medium with different working conditions. Specifically, the effect of pH, ionic strength, and temperature on SA-based films’ swelling was investigated. A heating magnetic stirrer (Arex, Velp Scientifica, Usmate Velate, Italy) controlled with an MGW Lauda R42/2 digital thermometer was used for temperature control. Specifically, the swelling measurements were performed, and data obtained under equilibrium conditions (joined after 60 s) were reported. The samples were removed from the solution, dried with filter paper to remove the excess water, and then weighed at time t (W_t_). The amount of adsorbed water was calculated as follows to obtain the Swelling % (Equation (1)):

(1) $$\mathrm{Swelling}\;\left(\%\right)=\frac{W_{t}-W_{0}}{W_{0}}\times 100$$

where W_t_ and W_0_ are the wet and dried film weights, respectively.

### 3.8. Determination of ABTS-Scavenging Activity

ABTS was solubilized in water to reach a final concentration of 7 mM. An ABTS radical cation (ABTS^•+^) was formed through the reaction of the ABTS stock solution with 500 µL of (NH_4_)_2_SO_4_ (0.6 mg/mL). The mixture was left in the dark at room temperature for 12 h before use. The measures were performed by diluting the latter solution (1:40) in phosphate-buffered solution (PBS) 0.1 M, pH 7.5, in the presence of appropriate amounts of the studied samples.

More specifically, at the beginning, SA and SA + GPWW films were completely solubilized in water to observe the maximum antioxidant action. For that purpose, the films were prepared without the treatment with CaCl_2_ solution (5%) to favor their complete dissolution. Samples of these solutions measuring 20 µL were tested by placing them in contact with the ABTS solution. The results are expressed in terms of % of ABTS bleaching and interpreted as a function of the antioxidant ability of SA and SA + GPWW films. Equation (2) was used to calculate the percentage:

(2) $$\%\;\mathrm{bleaching}=\frac{A_{\mathrm{ABTS}}-A_{\mathrm{sample}}}{A_{\mathrm{ABTS}}}\times 100$$

where $A_{\mathrm{sample}}$ is the absorbance value at 800 nm of the solution containing ABTS and the mentioned aliquot of samples at several incubation times, while $A_{\mathrm{ABTS}}$ is the absorbance value of the ABTS solution read at the same wavelength before the studied substrate was added.

### 3.9. Photostability of SA and SA + GPWW Composite Films

The SA-based film photostability was assessed by irradiating it with (i) a solar simulator lamp (Oriel Corporation, Stratford, CT, USA, Model 6684) equipped with a Xenon lamp (150 W) with an E_0_: 1482 mW/cm^2^~1.48 suns; (ii) a home-made Neon lamp (emission in the 400–700 nm range, nominal power 60 mW/cm^2^); and (iii) a UV lamp (Spectroline, Model CNF 280C/FE, λ 254 nm, light flux 0.2 mW/cm^2^; Melville, NY, USA). After irradiation for several time intervals, the samples were spectrophotometrically monitored.

### 3.10. Water Contact Angle (WCA) Measurements

The wettability of the samples was studied using static WCA measurements, performed through an automatic goniometer (Nord-test s.r.l., Alessandria, Italy) after depositing 2 µL of distilled water droplets. The contact angle values were obtained for SA and all SA + GPWW composite film typologies, averaging three measurements achieved in different parts of the same sample.

### 3.11. Water Vapor Transmission Rate (WVTR) Measurements

The WVTR of the samples was evaluated using a 7002 Water Vapor Permeation Analyzer (Illinois Instruments, Inc., Johnsburg, IL, USA). The instrument is equipped with a Pb_2_O_5_ sensor, and it displays the WVTR g/m^2^/day. According to Faraday’s laws of electrolysis, the electrolytic current is a measure of the rate at which water is electrolyzed. Under equilibrium conditions, this equals the rate at which the Pb_2_O_5_ film absorbs moisture. Thus, the absolute measure of the moisture in the sample is obtained using the gas flow rate through the housing and the current in the cell. The films were stored in the cell at 37 °C and under 90% relative humidity for 24 h.

## 4. Conclusions

In this work, water-resistant SA-based films are proposed as food packaging material with the goal of evaluating their role in preserving food in the future. Both internal and external gelation methods were adopted for their preparation. Indeed, the SA-based films obtained using the internal gelation approach were rapidly dissolved in water. In contrast, the films derived from the external approach appeared stable and durable for a long time in a water medium with a very low swelling % and a relatively high WCA. In particular, the effect of several parameters affecting the swelling process of these films, and thus their stability in water, was evaluated: the films were slightly temperature-responsive and were not affected by the change in pH. However, the materials appeared not stable if placed in contact with highly concentrated salt solutions. In particular, cations strongly competed with Ca^2+^ present in the alginate network, favoring the ionic exchange between the inner and outer sides of the film, weakening its network. According to the Bio-Circular Economy and Green Chemistry principles, the properties of these films were boosted by incorporating the GPWW extract inside the alginate matrix, which is particularly rich in polyphenolic compounds, to obtain SA + GPWW composite films. GPWW conferred antioxidant and UV-light-protection activity to SA. The ability of SA + GPWW films to protect SA and potentially the food itself from UV-light irradiation was successfully proposed. Indeed, the polyphenolic components in GPWW screened UV irradiation, preventing packaging degradation. By starting from these results, this work suggests the potential application of the proposed alginate-based films for food packaging. Work is in progress in our laboratory investigating the role of these substrates in retarding the biogenic amine production and microorganism growth in packaged food.

## Figures and Tables

**Table 1:** Contents of bioactive compounds (mg/L) in GPWW extract analyzed using HPLC-MS/MS.

No.	Compound	GPWW (mg/L)
	*Phenolic acids*	
1	Gallic acid	26.57
2	Neochlorogenic acid	n.d.
3	Chlorogenic acid	n.d.
4	p-Hydroxybenzoic acid	0.61
5	3-Hydroxybenzoic acid	n.d.
6	Caffeic acid	0.46
7	Vanillic acid	19.67
8	Syringic acid	3.46
9	p-Coumaric acid	0.37
10	Ferulic acid	0.02
11	3,5-Dicaffeoylquinic acid	0.01
12	Ellagic acid	3.54
	Flavonoids	
	(A) *Anthocyanins*	
13	Delphinidin 3,5 diglucoside	0.19
14	Delphinidin3-galactoside	1.91
15	Cyanidin-3-glucoside	0.87
16	Petunidin-3-glucoside	2.61
17	Pelargonidin-3-rutinoside	n.d.
18	Pelargonidin-3-glucoside	0.002
19	Malvidin-3-galactoside	16.76
	(B) *Flavonols*	
20	Rutin	0.05
21	Isoquercitrin	0.23
22	Quercitrin	0.01
23	Myricetin	0.54
24	Kaempferol-3-glucoside	0.02
25	Quercetin	6.92
26	Isorhamnetin	0.05
27	Hyperoside	0.42
28	Kaempferol	0.50
	(C) *Flavan-3-ols*	
29	Catechin	27.10
30	Epicatechin	9.93
31	Procyanidin B2	26.64
32	Procyanidin A2	0.17
	(D) *Dihydrochalcones*	
33	Phloridzin	0.09
34	Phloretin	0.003
	(E) *Flavanones*	
35	Hesperidin	n.d.
36	Naringin	n.d.
	Stilbenes	
37	Resveratrol	n.d.
	*Non-phenolic acids*	
38	Trans-cinnamic acid	0.04
	Total phenolic content	149.73

n.d., not detectable. All compounds’ relative standard deviation (RSD) ranged from 2.29 to 8.14%.

## Data Availability

All of the data is contained within the article and the Supplementary Materials.

## Supplementary Materials

The supporting information can be downloaded at https://www.mdpi.com/article/10.3390/ijms241411462/s1.
