# Supplementary material for: Realizing Eco-Friendly Water-Resistant Sodium-Alginate-Based Films Blended with a Polyphenolic Aqueous Extract from Grape Pomace Waste for Potential Food Packaging Applications
Source: Int J Mol Sci. 2023 Jul 14;24(14):11462. doi: 10.3390/ijms241411462 (PMC10380346; doi:10.3390/ijms241411462)
Supplement: Supplementary file 1 [file ijms-24-11462-s001.zip › ijms-2474388-supplementary.pdf]

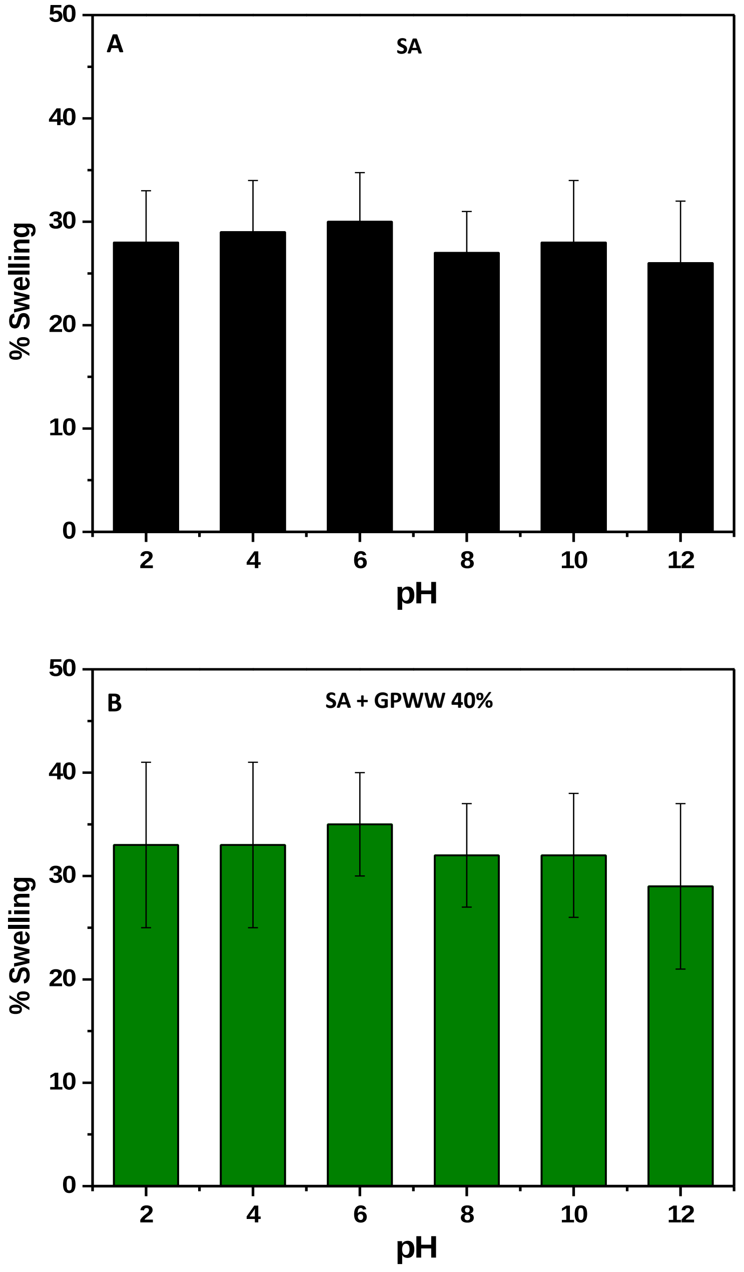

Figure S1: % of swelling related to SA (**A**), and SA+GPWW 40% (**B**) in water at different pH values.

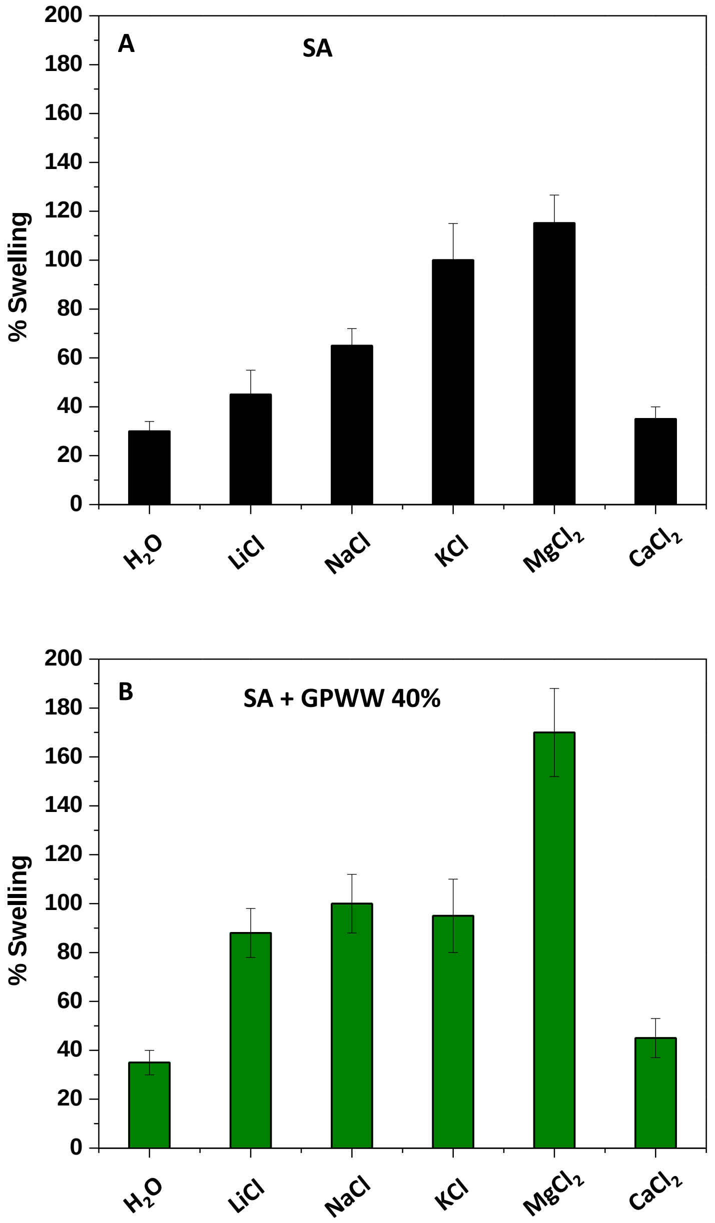

Figure S2: % of swelling related to SA (**A**) and SA+GPWW 40% (**B**) in salt solution evaluating the effects of anions and cations (having different sizes and charges).

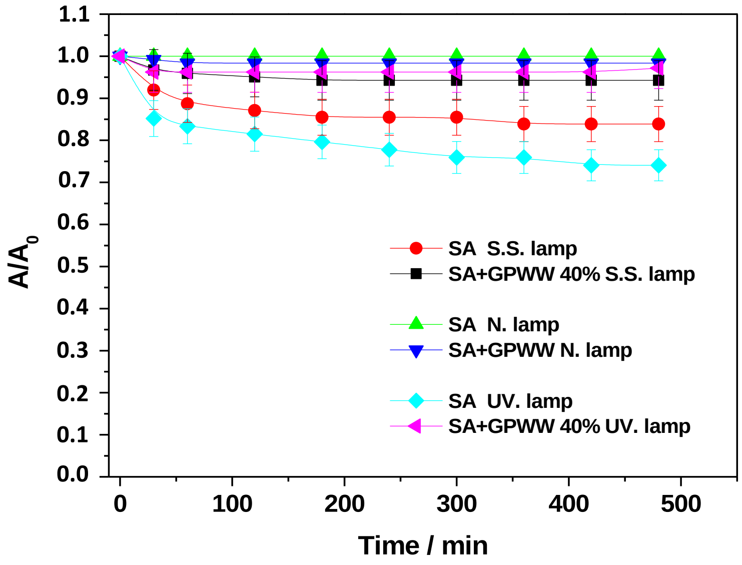

Figure S3: SA and SA+GPWW 40% photostability (S.S. lamp= Solar Simulator lamp; N. lamp = Neon Lamp) spectrophotometrically monitored.
